# Supplementary material for: Management of dyslipidemia after allogeneic hematopoietic stem cell transplantation
Source: Lipids Health Dis. 2022 Aug 2;21:65. doi: 10.1186/s12944-022-01665-3 (PMC9344644; doi:10.1186/s12944-022-01665-3)
Supplement: Supplementary file 1 — Additional file 1. [file 12944_2022_1665_MOESM1_ESM.pdf]

MS___________

WORD COUNT 6127
TIME SUBMITTED 18-MAR-2022 08:10AM
PAPER ID 83784806

18-Mar-2022 08:10AM

6127 words • 85 matches • 40 sources

FAQ

iThenticate

MS__________

Quotes Excluded

Bibliography Excluded

15%

Similar

Management of Dyslipidemia after Allogeneic Hematopoietic

Stem Cell Transplantation

Abstract

Dyslipidemia is one of the complications after allogeneic hematopoietic stem

transplantation (allo-HSCT), and it is often underestimated and undertre

Dyslipidemia in allo-HSCT recipients has been confirmed to be associated

endocrine dysfunction, acute and chronic graft-versus-host disease (aGVHD

Match Overview

Match

1

Bernard Lawrence Marini, Sung Won Choi, Craig Alan Byersdorfer, Simon Cro...

Crossref130 words

2%

2

Griffith, Michelle, Madan Jagasia, and Shubhada Jagasia. "Diabetes Mellitus Af...

Crossref80 words

1%

3

www.bloodjournal.org

Internet80 words

crawled on 03-Mar-2015

4

Reena Kumari, Senthilnathan Palaniyandi, Gerhard C. Hildebrandt. "Metabolic ...

Crossref59 words

1%

5

coek.info

Page: 1 of 30

Text-Only Report

Management of Dyslipidemia after Allogeneic Hematopoietic Stem Cell Transplantation

Abstract

Dyslipidemia is one of the complications after allogeneic hematopoietic stem cell transplantation (allo-HSCT), and it is often underestimated and undertreated. Dyslipidemia in allo-HSCT recipients has been confirmed to be associated with endocrine dysfunction, acute and chronic graft-versus-host disease (aGVHD and cGVHD), immunosuppressive agent application, etc. However, few studies have illustrated the accurate molecular signaling pathways involved in dyslipidemia, and there are no standard guidelines for dyslipidemia management after HSCT. This review will discuss the pathogenesis of dyslipidemia, especially the association with aGVHD and/or cGVHD. Comprehensive treatment methods for dyslipidemia after HSCT will also be summarized.

Introduction

Allogeneic hematopoietic stem cell transplantation (allo-HSCT) is an effective treatment method to reconstruct the normal hematopoiesis and immune function of patients. However, posttransplantation complications remain an obstacle for recipients to achieve long-term survival. Dyslipidemia after allo-HSCT is a common event, and it is defined as disorders of lipoprotein metabolism, including high total cholesterol (TC),

low-density lipoprotein cholesterol (LDL-C), triglycerides (TGs), and low high-density lipoprotein cholesterol (HDL-C) [1, 2]. In the general population, the incidence of dyslipidemia increases with age, and it can lead to a rising incidence of abdominal obesity, cardiovascular disease (CVD), and atherosclerotic cardiovascular disease (ASCVD) [3].

Previous studies have indicated that dyslipidemia after allo-HSCT shares some similar prevalent characteristics within the general population. A nonignorable question in clinical practice is that dyslipidemia is often underestimated and undertreated for transplantation recipients. It has been reported that the prevalence of dyslipidemia in transplant recipients ranges from 40% to 80% [4, 5], especially after kidney transplantation, which can be as high as 80% [5]. Oudin *et al*. analyzed 170 patients who received allo-HSCT for childhood leukemia. They found that the cumulative incidence of metabolic syndrome (MS) was 13.4% at 25 years and 35.5% at 35 years, which was significantly higher than that among the French population (4% and 5.6%, respectively) [6]. Another study confirmed that patients who underwent allo-HSCT were at higher risk of developing CVD more often and earlier than the age- and sex-matched healthy population [7]. Therefore, a better understanding of the prevalence and pathogenesis of dyslipidemia and the provision of effective prophylactic and therapeutic strategies are essential and urgent to improve the longevity and outcome of allo-HSCT recipients.

In recent decades, the most widely accepted etiologies of dyslipidemia after allo-HSCT have included age, sex, immunosuppressive agent application, and endocrine

dysfunction. Recently, some studies have proposed that there might be an interaction between dyslipidemia and acute and/or chronic graft-versus-host disease (aGVHD and/or cGVHD) [2]. However, literature that interprets the exact molecular mechanism and signaling pathways involved in dyslipidemia during the development of GVHD is rare. DeFilipp *et al*. published a comprehensive and practical guideline on screening and preventing MS and CVD in recipients following HSCT on *bone marrow transplantation* in 2017 [8]. This recommendation included multiple aspects, including lifestyle improvement, risk factor assessment, and the monitoring frequencies of the associated hematological parameters. However, it does not address many aspects of lipid-lowering medications. In this review, we summarized the pathogenesis of dyslipidemia and focused on the association with GVHD in patients who underwent allo-HSCT. The existing lipid-lowering treatments and promising target agents will also be discussed in this review.

Prevalence of dyslipidemia after HSCT

Compared with the healthy population, dyslipidemia is more common in allo-HSCT recipients. It has a younger age of onset in patients who have undergone HSCT, especially in allo-HSCT recipients [3]. Premstaller *et al*. reported that the prevalence of dyslipidemia in patients with hematopoietic diseases before transplantation was 36% and 28% in the autologous and allogeneic groups, respectively. This might be explained by the fact that patients who underwent auto-HSCT were older than those in the allo-HSCT group [9]. Dyslipidemia seemed to be a more severe event in allo-HSCT

recipients compared to other long-term complications. Compared to other complications, such as infection and GVHD, dyslipidemia has often been ignored by many clinicians because it usually develops slowly and insidiously.

The next question is which period is the most high-risk onset for dyslipidemia posttransplantation. Premstaller *et al*. concluded from a retrospective study that the prevalence rose to 62% and 74% 3 months after HSCT. At 25 years, it was 67% and 89% [9]. Bis *et al*. reported that the most common disturbances in the first half of the year after HSCT included low HDL levels (41.36%), increased levels of TGs (68.35% of the tested population) and TC (38.46%) [10]. HDL is often recognized as a protective factor against lipid metabolism. Consistent with the general population, female patients exhibited relatively higher HDL levels in the first 6 months than male patients after HSCT [10]. This difference might be explained by the fact that estrogens are considered a protective factor to retain the activity of the hydrolytic enzymes of lipids.

Overall, dyslipidemia is a more common complication post-transplantation than we realized, especially in allo-HSCT recipients. In this review, we will narrow our review scope to allo-HSCT recipients.

Etiology of dyslipidemia in patients after allo-HSCT

Oudin and his colleagues analyzed the potential mechanisms of MS. During chemotherapy or conditioning, cytotoxic drugs, such as alkylating agents, anthracyclines, antimetabolites, and vinca-alkaloids, can induce mitochondrial dysfunction, and endothelial cytotoxicity leads to insulin resistance, steatosis, and

hypertension [11]. Furthermore, noncytotoxic drugs, such as steroids, can cause hyperglycemia and dyslipidemia [3]. Their meaningful but incomplete investigation helped us have a better understanding of dyslipidemia. In addition to the common reasons within the general population, such as age, obesity, diet, less physical activity, and menopause, some specific factors can also lead to or aggravate dyslipidemia in allo-HSCT recipients (Table 1).

Table 1 Etiology of dyslipidemia in patient after allo-HSCT

**Causes**	**Mechanism**		**Dyslipidemia profile**
IR and diabetes [12-14]	In high glucose internal environment, LDL-C enzyme activity in adipose tissue decreases significantly, TGs metabolism slows down accordingly.		VLDL, TGs, and TC ↑
Immunosuppressants [15-17]	Glucocorticoids	① Reduce GLUT2 and glucokinase expression, stimulate B-cell apoptosis, enhance skeletal muscle insulin resistance, and reduce insulin uptake and glycogen storage. ② Enhance IR. ③ Lead to overproduction of triglycerides and VLDL-C. ④ The clearance of triglyceride-rich lipoproteins such as chylomicron and VLDL-C is reduced by corticosteroid-induced LPL.⑤enhances esterase activity and decreases LDL receptors. ⑥ lead to weight gain.	VLDL, TGs, and TC ↑
	CNI	① Inhibit steroid 26-hydroxylase, thereby inhibiting cholesterol conversion into bile acid, blocking LDL receptors, leading to	VLDL and LDL ↑

		serum LDL elevation, and affecting VLDL and LDL clearance by changing lipase activity. ② Interfere with the binding of LDL-C to LDL receptors and promote hepatic lipase activity and the decrease of LPL. ③ inhibit insulin secretion of pancreatic B cells by binding to cyclin-D and inducing b cell apoptosis.	
	mTOR inhibitors	① Expand the FFA pool and increase hepatic VLDL synthesis. ② Increase lipolysis via augmentation of hormone-sensitive lipase (increasing circulating FFA), interfering with triglyceride metabolism, decreasing triglyceride storage, and disrupting the insulin-signaling pathway.	VLDL, TGs ↑
GVHD [1, 18, 19]	① Induce intrahepatic cholestasis and nephrotic syndrome. ② T cells in the process of GVHD depend on lipid biosynthetic pathways to meet the high metabolic requirement of rapid clonal expansion. ③ Disturbance of intestinal microbiota.		TC, and TGs ↑
Intestinal microflora	Gastrointestinal microflora can alter the enzymes that participate in the metabolism of three substances.		Multiple metabolic disorders
Nutrition intake	Excessive high-sugar and high-fat nutrient intake.		TC, and TGs ↑
Others [20, 21]	Hypothyroidism, hyperthyroidism, hypogonadism, and growth hormone deficiency. Most of them are associated with conditioning such as TBI or cytotoxic agents.		Multiple metabolic disorders

Insulin resistance (IR) and diabetes

Previous studies have reported a prevalence of type 2 diabetes mellitus (T2DM), impaired glucose tolerance, and IR in allo-HSCT recipients of 17%, 26%, and 52%, respectively [12]. **Error! Reference source not found.** An Israeli study reported that 3.3% of survivors, at a mean of 6.2 years after receiving a bone marrow transplant (BMT) in childhood or adolescence, developed T2DM. However, the prevalence of T2DM in general adolescence is only 0.0029% in Europe [13]. A multiple risk factor analysis indicated that total body irradiation (TBI), immunosuppressants (such as glucocorticoids), and calcineurin inhibitors (CNIs) were closely associated with T2DM or IR [13]. TBI is popularly used in either myeloablative or nonmyeloablative conditioning. Traggiai *et al*. reported posttransplant diabetes (PTDM) in 6 of 74 BMT patients (8%) who received TBI and 0 of 11 patients who did not receive TBI. In a study of 34 survivors of auto- or allo-HSCT procedures in childhood, IR was significantly more prevalent in patients who received TBI than in patients who received thoracoabdominal irradiation, patients not receiving irradiation, and healthy control subjects [14]. The association between IR and immunosuppressants will be discussed in the following part of this review.

IR can lead to impaired de novo fat synthesis and dyslipidemia. IR in the liver leads to the export of free fatty acids (FFAs) to the muscles and, in turn, is aggravated by IR. In the case of IR, muscle tissue obstructs sugar utilization. Enhanced activity of hormone-sensitive lipase results in many fatty acids being released from the fat tissue. For patients with T2DM, such free nonesterified fatty acids cannot be converted into

ketone bodies but act as raw materials to increase the synthesis of very low-density lipoprotein(VLDL), TGs, and TC in the liver. Therefore, patients with abnormal glucose tolerance or DM before or after transplantation are at a higher risk of dyslipidemia [11, 12, 14, 22].

Immunosuppressants

Many immunosuppressants, such as glucocorticoids, CNIs, and mammalian target of rapamycin (mTOR) inhibitors, affect both TC and TGs metabolism in a dose-dependent manner [3]. CNIs (such as cyclosporine and tacrolimus) have been popularly used in immunosuppressive therapy (IST) since they were approved by the Food and Drug Administration (FDA) in 1983. In a trial, 36 transplant patients treated with cyclosporine for just 2 months resulted in increases of average total cholesterol and LDL of 21% and 31%, respectively [15]. Cyclosporine (CSA) is associated with impaired glucose tolerance in approximately 35% of kidney transplant patients. Sirolimus (SRL), a classic mTOR inhibitor, also exhibits a significant adverse effect on lipid metabolism [16]. Morrisett *et al*. found that treatment with SRL resulted in an expansion in hepatic VLDL synthesis. Fuhrmann *et al*. observed the effects of treatment with CSA or SRL on glucolipid metabolism in Wistar rats [17]. Their study showed significantly increased TGs levels in the serum, liver, and skeletal muscle resulting from tissue steatosis caused by the drug [17].

Despite the potential immunosuppressive effect, these immunosuppressants are a "double sword" when treating GVHD. Furthermore, most of them share a similar

metabolic pathway through the cytochrome P450 3A4 (CYP3A4) system with many lipid-lowering agents, including statins, by decreasing the clearance of statins. Therefore, we should pay more attention to the cross-linking effect when we simultaneously use immunosuppressants and statins.

GVHD

In addition to the most concerning target organs/systems of aGVHD or cGVHD, such as the skin, liver, intestinal tract, lungs, and bone marrow, lipid metabolism is also a critical target system of GVHD. However, few studies have reported the detailed mechanism of dyslipidemia during the process of GVHD. The relationship between dyslipidemia and GVHD is often underestimated until some researchers found a decreased frequency of aGVHD if statins were given to patients simultaneously [23]. In 2012, Kaguya *et al*. characterized the prevalence and risk factors for dyslipidemia in allo-HSCT recipients and found that almost half of them had hypercholesterolemia or hypertriglyceridemia [2]. Monica et al. conducted a retrospective analysis of 121 allo-HSCT recipients. Among them, 25 (22%) patients with aGVHD presented high hypercholesterolemia [18]. Previous researchers have proposed that there might be a mutually causal role between dyslipidemia and GVHD. However, the accurate interactive mechanism of these two abnormal biological processes is uncertain.

To better explore the association between the abnormal immune response and GVHD, T-cell activation and metabolic reprogramming have been the “hot points” in recent years [19]. It is well known that T cells play an essential role and are the primary effector

cells during the whole process of aGVHD or cGVHD. T-cell activation is the first and most important step to drive an immune response, attacking the host immune system by mounting potent cytokines. The metabolic changes of T cells are shown in a dynamic model. Initial T cells only rely on oxidative phosphorylation (OXPHOS) to meet their energy requirements [24]. After antigen recognition, initial T cells differentiate into effector T cells (Teff), and the primary energy source of Teff metabolism is converted to anaerobic glycolysis. Anaerobic glycolysis produces only 2 mol of adenylate triphosphate (ATP) per gram of glucose through independent mitochondrial metabolism, oxidizing to pyruvate to produce lactic acid. However, OXPHOS may produce up to 30 ATPs per glucose molecule [19]. T cells from patients with aGVHD polarize toward proinflammatory T cells and have higher glycolytic activity than those from patients who do not have aGVHD. Compared with resting T cells, T cells involved in the process of aGVHD have to increase their oxygen consumption to meet the energy requirements, exhibit hyperpolarized mitochondrial membrane potential, and increase reactive oxygen species (ROS) production. In turn, the release of ROS enhances T-cell activation and expansion, resulting in the development of aGVHD [19]. Inhibition of mTOR with rapamycin reduces glycolysis and enhances fatty acid oxidation (FAO) in donor T cells, reducing alloreactive T cells and enhancing regulatory T-cell (Treg) function; the latter has lower glycolytic activity [25]. In brief, glycolysis is a major metabolic pathway after T-cell activation, and Tregs primarily use the fatty acid and pyruvate oxidation (mitochondrial oxidation) pathways. Therefore, reducing glycolysis might be feasible to interrupt the process of T-cell activation and improve aGVDH.

Similar to aGVHD, dyslipidemia is also common during cGVHD. Previous studies have confirmed that cGVHD in the liver is usually accompanied by significantly increased levels of TC and TGs in the serum [2]. Yoshihiro *et al*. reported an increase in lipoprotein X (LP-X) in recipients [1]. LP-X is separated from LDL and is mainly composed of free cholesterol and phospholipids. The presence of LP-X in liver disease is regarded as the most sensitive and specific biochemical marker of cholestasis. Furthermore, LP-X may play a crucial role in the development of cholestatic hypercholesterolemia because it cannot inhibit the synthesis of new cholesterol in the liver [26]. LP-X increased in patients with cholestasis and was closely related to biliary obstruction. Thereby, bile salts and cholesterol cannot be removed through the bile duct, leading to gallbladder disease and decreased hepatiltriglyceridase (HTGL) activity [1]. HTGL decompose the TGs and cholesterol esters in cells into FFAs. A low level of HTGL is closely associated with hypertriglyceridemia and hypercholesterolemia. Impaired liver function and destruction of fat cells via autoimmunity after the onset of aGVHD are also causes of hyperlipidemia [2, 27]. In some rare situations, the manifestation of cGVHD involving the kidney is nephrotic syndrome, thus resulting in dyslipidemia.

Overall, the relationship of GVHD and dyslipidemia has attracted great attention to better understand their interaction mechanism, occurrence sequence, and therapeutic target for GVHD. A schematic diagram of dyslipidemia and GVHD is shown in Figure 1.

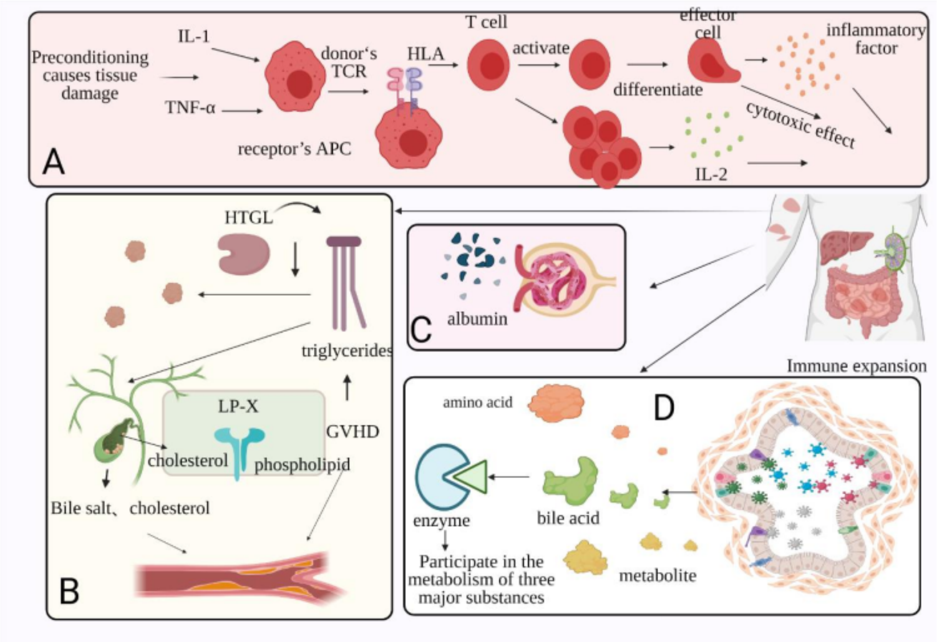

Figure1 Relationship between dyslipidemia and GVHD (designed with BioRender). A: Preconditioning results in tissue damage, releasing of various inflammatory factors, expansion, and differentiation of T cells. B: GVHD leads to a decrease in serum HTGL content, makes triglyceride fail to break down, and ultimately develops hypertriglyceridemia. Increased LP-X content leads to cholestasis, blocks cholesterol excretion, and eventually leads to hyperlipidemia. C: Extensive immune expansion can result in hypoproteinemia and hyperlipidemia. D: GVHD alters the intestinal microenvironment and affects the synthesis of enzymes that regulate substances.

Intestinal microflora

26

The intestinal flora plays a vital role in maintaining immune homeostasis, regulating intestinal function, and regulating metabolism. The intestinal flora is a complex and colossal ecosystem called the "second gene pool" of human beings—approximately

10^{14} bacteria in the normal human intestinal tract [28], with more than 800 types [29]. Intestinal inflammation can alter the diversity and composition of intestinal microorganisms, creating a state of dysbiosis [30]. A previous study confirmed that the gut microbiome is related to overweight, dyslipidemia, and IR [31]. Margaret *et al*. summarized microbial metabolites associated with hyperlipidemia, such as short-chain fatty acids (SCFAs), bile acids, and trimethylamine-N-oxide (TMO) [30]. SCFAs can activate the cyclic adenosine phosphate (AMP)/protein kinase A (PKA)/cyclic adenosine phosphate (cAMP) reaction element-binding protein pathway in the liver, enhancing oxidative metabolism, inhibiting liver fat production, and improving lipid levels. SCFAs can also stimulate the secretion of glucagon-like-1 (GLP-1) and gastroenteric peptide YY (PYY) and reduce the occurrence of hyperlipidemia [31]. TMO downregulates the enzymes CYP7A1 and CYP27A1 that are involved in bile acid synthesis, which might be mediated by the activation of the farnesoid X receptor and small heterodimer partner [30]. Intestinal flora can upregulate the element-binding protein-3 gene, promote cholesterol 7α-hydroxylase (CYP7A1) expression, stimulate bile acid synthesis, and reduce de novo fat synthesis in the liver. The combined action of choleidochrome acid and TGR5 can increase the level of cAMP, stimulate the secretion of type II deiodinase, increase thyroid hormone, promote the consumption of brown adipose tissue and increase heat production, thus improving lipid metabolism and preventing the occurrence of hyperlipidemia and other diseases [32].

In allo-HSCT recipients, the gastrointestinal tract can be damaged, resulting in intestinal microbiota dysbiosis due to the conditioning process (chemotherapy or

radiotherapy) or intestinal infection post allo-HSCT [33]. Disturbances of intestinal microbiota may be associated with the development and progression of infectious or noninfectious inflammation, including aGVHD. As a common target organ in aGVHD, intestinal tissue damage can, in turn, aggravate dysbiosis, resulting in an alteration of the enzyme profile in the metabolism of sugar, fat, and proteins [30].

Posttransplant nutrition and living habits

During conditioning and before hematopoietic reconstitution, the diet of the patients is restricted to maximally conserve digestive and absorptive functions and avoid potential intestinal infection. Even after hematopoietic reconstitution, recipients are also at a high risk of various pathogen infections due to the applications of immunosuppressants and immunodeficiency [34]. Severe mucositis or gastrointestinal complications of GVHD allow many patients to only receive partial or complete parenteral nutrition [35]. Gabriela *et al*. followed up 198 pediatric patients from 1995 to 2016, with a median follow-up of 3.8 years after HSCT. They concluded that many children required total parenteral nutrition due to severe mucosal toxicities or gastrointestinal complications of aGVHD. The return to the regular oral diet is prolonged and difficult [35]. The input of a large amount of high-sugar and high-fat nutrient solutions dramatically increases the TC and TGs levels in the blood of patients.

Most recipients cannot return to daily exercise in time due to poor performance status and multiple transplant-related complications. Psychological or social factors constrict

them from initiating activity, even though their physical condition has recovered well. Yu, J *et al*. analyzed 686 consecutive patients with acute leukemia who received allo-HSCT between January 2008 and December 2017. Their results showed that 56.4% of patients had standard body mass indices, 17.3% were underweight, 20.4% were overweight, and 5.8% were obese [36]. A previous study reported that one-quarter of relatively younger survivors smoke after HSCT, further deteriorating lipid metabolism. Overall, increased comorbidity was associated with a healthier lifestyle, resulting in the development of dyslipidemia [37].

Others

Genetic factors are also involved in dyslipidemia. Primary hypothyroidism has been reported in 10% to 50% of allo-HSCT recipients. Secondary autoimmunity from graft donors may also cause autoimmune thyroid disease in allo-HSCT recipients, leading to hypothyroidism or hyperthyroidism, especially in HLA-related donors. Hypothyroidism, elevated blood pressure, and hyperlipidemia occur in 45% of patients after liver transplantation [20]. Furthermore, hypogonadism and growth hormone deficiency may predispose to IR, MS, and dyslipidemia. This conclusion is consistent with the fact that sex dysfunction (SD) and hormone deficiency increase with age in the general population. Previous studies have confirmed that the age of SD and dyslipidemia is far younger in sex-matched allo-HSCT recipients [21].

Management of hyperlipidemia after allo-HSCT

To date, there are no widely recognized guidelines for dyslipidemia management post-HSCT. Clinicians usually rely on procedures for managing dyslipidemia after solid organ transplantation. The most commonly used measures to prevent and intervene in dyslipidemia include dietary control, lifestyle improvement, and drug intervention. The guidelines recommend the monitoring content, testing frequencies, and suggested agents for the treatment of hyperlipemia. Recently, new lipid-lowering drugs, such as proprotein convertase subtilisin/kexin type 9 (PCSK9) inhibitors, have been confirmed to be safe, effective, and tolerant in these populations. Here, we summarize the current therapeutic options for the prophylaxis or treatment of dyslipidemia in HSCT recipients.

General treatment

Like the general population, HSCT recipients should abandon bad habits such as alcoholism and smoking and taste preferences for salt, fat, and sugar. With guidance from the nutrition specialist, recipients should control total energy intake to meet essential daily nutrient requirements, allocate the proportion of nutrient elements, control weight, and perform regular and moderate metabolic exercise [36].

Changing the immunosuppressant regimen

Withdrawal of the immunosuppressants cannot be conducted in most situations. However, it might be feasible to adjust the dose or type of immunosuppressant. Anti-CD25 monoclonal antibody has been popularly accepted by clinicians to improve steroid-refractory or steroid-resistant aGVHD (SR-aGVHD), especially when the liver and intestinal tract are involved. Recently, Janus kinase inhibitors, such as ruxolitinib,

have been approved by the FDA for application in the treatment of SR-aGVHD. When used as a single agent or combined with calcineurin inhibitor (CNI) reduction, these immunosuppressive agents rarely cross-react with statins [38]. Many new anti-GVHD choices, such as mesenchymal stem cells (MSCs), integrin inhibitors, cytokine modulators, and brentuximab vedotin, have been investigated in multiple clinical trials. Although CNIs cannot be replaced, we might explore more effective and safe combination strategies to avoid adverse effects on lipid metabolism.

Lipid-lowering drugs

At present, the most commonly used lipid-lowering agents are statins, ezetimibe, fibrates, and niacin. In recent years, new lipid-lowering drugs, such as PCSK9 inhibitors, have been gradually applied to the clinic. The lipid-lowering mechanisms, main side effects, and drug interactions of these agents are listed in Table 2. Lipid-lowering drugs and their binding targets are shown in Figure 2.

Table 2 Lipid-lowering drugs after allo-HSCT

**Lipid-lowering drugs**	**Mechanism**	**Side effect**	**Drug interaction**
Statins [20, 39-41]	①Competitively inhibit the conversion of seemed to be more severe to HMG-COA reductase.	Elevated transaminases, myositis, and rhabdomyolysis.	Cyclosporine can increase the serum level of statins through effects on the cell membrane transporter multidrug-resistant protein 2.

	②Block important isoprenoid intermediates in the cholesterol biosynthetic pathway. ③Reduce and regulate immune function. (Reduce T cell activation and co-stimulatory molecules on APCs by reducing MHC-II. Increase levels of interleukin 10, an anti-inflammatory cytokine with TH2 phenotypic characteristics) ④Others: improve endothelial function, enhance and stabilize atherosclerotic plaques, reduce oxidative stress and inflammation, and inhibit thrombosis response.		Inhibitors of CYP3A4 such as azole antifungals, non-dihydropyridine calcium channel blockers (verapamil and diltiazem), and macrolide antibiotics increase the risk of toxicity of statins.
Ezetimibe [42, 43]	Reduces intestinal cholesterol absorption by inhibiting small intestinal cholesterol transporter, lowers plasma cholesterol level and liver cholesterol reserves by selectively inhibiting small intestinal cholesterol transporter.	Elevate transaminases	Increase cyclosporine levels.

Fibrates [44]	Increase the expression of apolipoprotein genes linked to the stimulus, enhance lipoprotein lipase activity	Cholelithiasis Gastrointestinal upset Myopathy	The risk of myopathy is increased when fibrates are given with statins, particularly in patients with impaired kidney function or those on cyclosporine.
Niacin [39]	① Inhibits glycerin esterase activity in adipose tissue. ②Enhance LPL activity, promote the hydrolysis of plasma TGs.	Exacerbate hyperglycemia and hyperuricemia, flushing, and gastrointestinal intolerance, enhance the blood pressure	Lower effect of ganglion blockers, calcium channel blockers, and adenoid inhibitors
PCSK9 inhibitors [45, 46]	Binds to LDLR, mediates LDLR to enter liver cells, and is finally degraded by lysosomes	Respiratory tract infection	——

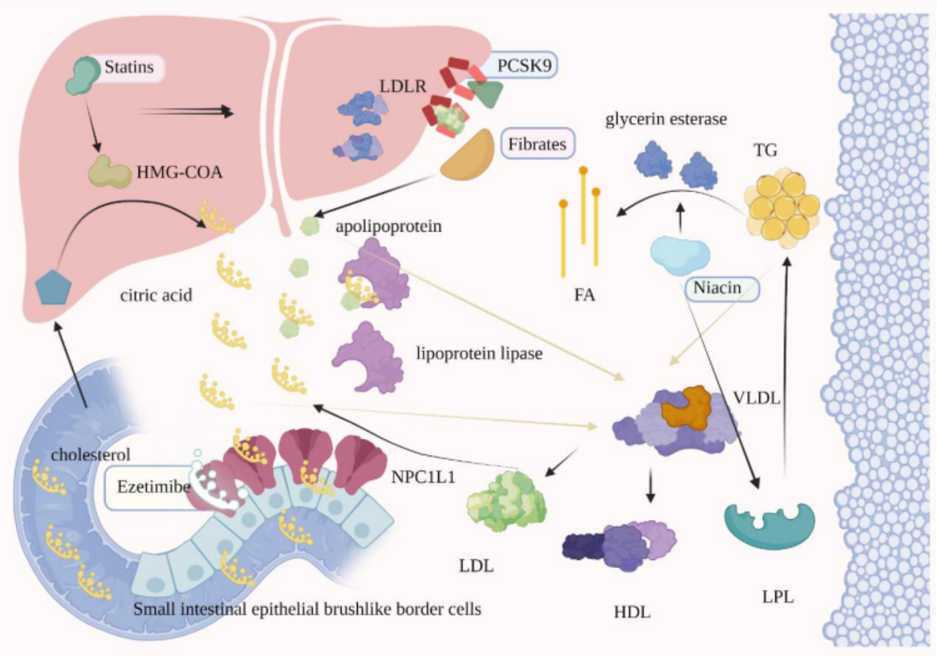

Figure2 Targets of lipid-lowering drugs (designed with BioRender).

Statins

Statins are currently the most popularly used lipid-lowering drugs. Statins inhibit the conversion of 3-hydroxy-3-methylglutaryl-coenzyme A (HMG-CoA), transforming HMG-CoA into mevalonic acid. Stains reduce the production of cholesterol in the liver and lead to upregulation of LDL receptors, thereby increasing LDL clearance. Statins can lower LDL cholesterol in a range of 25% to 60% and reduce lower TGs in a range of 10% to 37% in the general population [20]. Therefore, statins might be beneficial for patients with hyperlipidemia posttransplant, especially those who have higher LDL levels.

Most statins, except for fluvastatin and pravastatin, are mainly metabolized through the CYP3A4, CYP2C8, and CYP2C9 pathways, as are CNIs and mTOR inhibitors. As

a classic CYP3A4 inhibitor, CSA can increase the serum level of statins by affecting the cell membrane transporter multidrug-resistant protein 2. Thus, CSA can potentially increase the risk of toxicity of statins. Other inhibitors of CYP3A4, including nondihydropyridine calcium channel blockers, azole antifungals, and macrolide antibiotics, could cause the abnormal metabolism of statins. It is worth noting that, although pravastatin is also metabolized by the liver, it is not metabolized by the CYP enzyme system, so it has relatively few interactions with other drugs [47]. Another statin with fewer drug interactions is fluvastatin. It is mainly metabolized by CYP2C9 the pathway (approximately 75%), with a lower percentage being metabolized by the CYP2C8 and CYP3A4 pathways [41]. Therefore, these two statins (especially pravastatin) can be the first-line choice when combined with other CYP3A4 inhibitors because they have fewer drug-to-drug interactions. It is important for clinicians to choose relevant safe lipid-lowering drugs for allo-HSCT recipients. Rare but important SEs of statins include myositis, elevated transaminases, and rhabdomyolysis [39]. In the general population, transaminases should be measured within 8-12 weeks of statin use and within 2-4 weeks for patients with GVHD. To date, there have been only a few small-scale studies reporting statins applied in allo-HSCT recipients with stable cGVHD of the liver [48]. Statins should be avoided in patients with decompensated cirrhosis, severely elevated liver enzymes (more than 3x elevated transaminases), or acute liver failure [48].

In addition, previous studies have confirmed that statins have immunomodulatory effects and might be beneficial in controlling GVHD. Statins indirectly reduce T-cell

activation and costimulatory molecules on antigen-presenting cells (APCs) by reducing major histocompatibility complex II (MHC-II) expression [40].**Error! Reference source not found.** Lovastatin and simvastatin have been shown to inhibit leukocyte functional associated antigen-1 (LFA-1). The latter is an integrin molecule involved in regulating lymphocyte transport and activation. T-cell activation induces conformational changes in LFA-1, which binds to ligands on APCs with high affinity, leading to T-cell transport and further activation. The allosteric combination of simvastatin and lovastatin with LFA-1 forces the latter to maintain a low association, thus reducing GVHD incidence. In previous experimental data, researchers have found that the concentration of interleukin-10 (IL-10), an anti-inflammatory cytokine with T helper 2 (TH2) phenotypic characteristics, was elevated in statin users. The above preliminary or clinical investigations support the possible mechanism of immune regulation by statins [40].

Based on the exciting results of statins in improving aGVHD in animal models, some researchers have tried to apply statins in the prophylaxis and treatment of aGVHD [23]. However, the protective effect of statins on the prophylaxis and treatment aGVHD is still controversial. Here, we chose two representative studies and compared their results in Table 3 [23, 49]. Taken together, the preliminary multipotent effects of statins indicated both lipid-lowering and immunomodulatory effects. We must admit that it is too early to draw a definite conclusion whether statins can truly prevent aGVHD before more multicenter, randomized, double-blind, rigorously designed clinical trials have been performed.

Table 3 Comparison of atorvastatin for the prophylaxis of aGVHD based on two representative studies

	**Study 1 (Ref.49)**	**Study 2 (Ref.23)**
First author	Yvonne A.Efebera	Mehdi Hamadani
Journal	*Biology of Blood and Marrow Transplantation*	*Journal of Clinical Oncology*
Publication year	2016	2013
Number of participants	50 patient-donor pairs	30 patient-donor pairs
Median age (recipient)	51	54.5
Median age (donor)	50	52.5
Control group	Historical control subjects(n=96)	Historial data from the same institution
Conditioning regimen and type		
Myeloablative conditioning	Fludarabine/busulfan (15%)<br>TBI/etoposide (10%)<br>TBI/cyclophosphamide (5%)	Busulfan/cyclophosphamide (6.6%)<br>Fludarabine/busulfan (36.6%)
Reduced-intensity conditioning	Fludarabine/busulfan (65%)<br>Fludarabine/melphalan (5%)	Fludarabine/busulfan (56.6%)
Diagnosis	AML (30%)<br>NHL+HL (36.6%)<br>MDS (10%)<br>PMF (6.6%)<br>Others (16.6%)	AML (33%)<br>NHL+HL (15%)<br>ALL (15%)<br>CML(8%)<br>MDS/CMML (18%)<br>Others (13%)

Study time	March 2012- January 2014	September 2010- October 2012
GVHD prophylaxis	Methotrexate and tacrolimus	Methotrexate and tacrolimus
Median CD34 cell × 10^6 /kg infused	5.90	4.25
Median CD3 cells × 10^8 /kg infused	2.65	3.30
Dose of atorvastatin	40 mg/day	40 mg/day
Duration of medication	Beginning 14 days before the anticipated start of stem cell collection	Starting 14 to 28 days before the anticipated first day of stem-cell collection
Infection	Cytomegalovirus and Epstein-Barr virus reactivation, BK viremia, and fungal or bacterial infections were not significantly different between groups	Three patients (10%) each developed cytomegalovirus reactivation and BK-viral hemorrhagic cystitis
Immune reconstitution	Less NK cells and Tregs than the non statin group	No statistically significant change
Cytokines	Elevation of RANTES	A trend toward higher mean IL-10 concentrations
Incidences of grades II to IV aGVHD at days +100 and + 180 (%)	30% and 40%, respectively	3.3% and 11.1%, respectively.
Incidence of II°-IV°aGVHD in the control group at days +100 (%)	28%.	Ranged from 30% to 35%.
1 year NRM (%)	5.5%	9.8%
1 year PFS (%)	54%	65%
1 year OS (%)	82%	74%

Conclusion	Negative	Positive

Ezetimibe

Ezetimibe is the first cholesterol absorption inhibitor. Ezetimibe effectively reduces intestinal cholesterol absorption and lowers plasma cholesterol levels and liver cholesterol reserves by inhibiting small intestinal cholesterol transporters. Ezetimibe can increase CSA levels, so some experts recommend lower than regular ezetimibe doses (no more than 5 mg per day) and strict monitoring of CSA levels [42]. Drug-to-drug interactions should also be considered when ezetimibe is combined with CSA by the extra-CYP3A system. Ezetimibe does not induce cytochrome P450 drug-metabolizing enzymes and shows no significant interactions with drugs known to be metabolized by cytochrome P450. However, the precise interaction mechanism of ezetimibe and CSA is unclear.

Fibrates

Fibrates are a kind of peroxidase scion activated receptor (PPAR) alpha agonist and 3 are generally used for severe hypertriglyceridemia (>500 mg/dL TGs). They can increase the expression of apolipoprotein genes linked to the stimulus and enhance lipoprotein lipase activity, thus reducing TGs levels, elevating HDL-C levels, and delaying the development of atherosclerotic plaques [44]. Previous studies have confirmed that fibrates can reduce plasma TGs, LDL-C, and HDL-C levels by at least 30%, 15%, and 5-15%, respectively. Potential SEs include cholelithiasis, gastrointestinal disorders, and myopathy. Patients are more likely to develop myopathy when taking statins or CSA.

Gemfibrozil has been shown to increase statin levels by 1.9-5.7 times by inhibiting glucosidation. A better treatment option is to combine fenofibrate with statins [47].

Niacin

Evidence for niacin in allo-HSCT populations is limited. However, niacin is not associated with significant pharmacokinetic drug interactions, making it a desirable option for patients on CSA or azole antifungal agents. A large dose of niacin substantially affects lipid regulation by reducing lipid production and promoting lipid decomposition [39]. Niacin can be prescribed to patients with elevated cholesterol and TGs levels. It can lower LDL-C and TGs levels by approximately 16% and 38%, respectively, and raise HDL-C levels by 22%. Niacin inhibits glycerin esterase activity in adipose tissue and fatty tissue mobilization, thus reducing the synthesis of VLDL in the liver. Niacin can also enhance lipoprotein lipase (LPL) activity, promote the hydrolysis of plasma TGs, reduce VLDL concentration, and reduce VLDL conversion to LDL-C, thus reducing TC and LDL-C levels.

Notably, niacin shows anti-inflammatory activity through its activity on the g-protein double receptor GPR109A (carboxylic acid receptor 2). GPR109A is expressed in adipocytes and can suppress the release of FFAs. Janet E. Digby *et al.* found the substantial anti-inflammatory effects of niacin, including decreased chemokine and cytokine production, suppressed signaling through the NF-κB pathway, and reduced adhesion to activated endothelial cells, according to effects on very late antigen-4 (VLA-4) [50]. This effect supports niacin as a potential candidate to control GVHD.

PCSK9 inhibitors

In recent years, monoclonal antibodies against PCSK9 have been confirmed to be safe and effective lipid-lowering treatment. PCSK9 is a member of the protease K subfamily of the proprotein invertase family. It encodes 692 amino acids and is primarily expressed in the liver and ileum. PCSK9 is synthesized and secreted by the liver, binds to LDLR, mediates the entry of LDLR into liver cells, and finally degradation by lysosomes [51]. PCSK9 inhibitor was introduced for clinical application in 2009. The commonly used clinical PCSK9 inhibitors are evolocumab and alirocumab. Warden *et al*. found that evolocumab therapy was associated with a 60% reduction in LDL-C, from a baseline of 92 mg/dL to a median of 30 mg/dL [45]. Evolocumab can also induce a 15% relative risk reduction (RRR) in the primary 5-point major adverse cardiovascular events (MACE) endpoint (cardiovascular death, myocardial infarction, stroke, hospitalization for unstable angina, or coronary revascularization), during a median follow-up of 2.2 years [45]. At present, the most common clinical adverse effect (AE) is induration at the injection site. Feingold *et al*. found that infection and inflammation stimulate the expression of PCSK9 in the liver, thus reducing the level of LDL receptor (LDLR) protein in the liver and leading to an increase in circulating LDL levels [52]. PCSK9 inhibitors reduce LDL-C by reducing the degradation of the LDL receptor.

Meanwhile, increasing evidence has shown a close relationship between PCSK9 and inflammation [52]. PCSK9 was confirmed to induce an inflammatory response in macrophages by releasing proinflammatory cytokines, such as tumor necrosis factor α

(TNF-α), IL-1, and IL-6. Tang *et al*. demonstrated that PCSK9 enhances the secretion of inflammatory cytokines by activating the TLR4/NF-κB pathway in macrophages **Error! Reference source not found.**. ROS are closely related to inflammation according to previous studies. Ding *et al*. found regenerative feedback in ROS generation and PCSK9 expression. In their experiment, PCSK9 knockout (KO) mice showed significant suppression of NADPH oxidase (p47phox and gp91phox subunits) in aortic tissues, and $p47^{phox}$ and $gp91^{phox}$ KO mice showed an almost 50% reduction in serum PCSK9 levels. Administration of exogenous hPCSK9 to WT mice resulted in a 50% increase in NADPH oxidase in aortic tissues [53]. Therefore, PCSK9 inhibitors might have both anti-inflammatory and anti-GVHD effects. Different from statins, PCSK9 inhibitors show no obvious SEs and no apparent drug-to-drug interactions, which makes them an excellent promising lipid-lowering choice in allo-HSCT recipients[40]. To date, there is clinical data for the use of PCSK9 inhibitors in allo-HSCT recipients. We only found three clinical trials (NCT03537742, NCT04665830, and NCT04608474) on PCSK9 inhibitors (one on alirocumab and two on evolocumab) in heart and renal transplant patients. **Error! Reference source not found.**. In our center, we have administered evolocumab to several recipients after allo-HSCT who developed cGVHD involving the liver and had dyslipidemia. Our preliminary clinical data indicated the efficacy and safety of evolocumab with regard to its lipid-lowering effect, with decreases in TGs, TC, and VLDL levels; recovery of liver function; and improvement in cGVHD, as we anticipated. Overall, the relation-ship between PCSK9 inhibitors and GVHD needs to be validated in more preclinical and clinical

investigations. As a promising lipid-lowering agent, we anticipate more positive results to give us more evidence in allo-HSCT re-cipients.

Comparisons with other studies

Previous studies have summarized risk factors for dyslipidemia in allo-HSCT recipients, such as sex, age, obesity, and diabetes. This review summarizes the mechanism of dyslipidemia involved in the development of GVHD with several aspects, including immunosuppressive application and intestinal flora. We also emphasized the potential use of statins and PCSK9 inhibitors in both lipid-lowering and anti-GVHD efficacy.

Strengths and limitations of the review

To date, few studies have interpreted the exact molecular mechanism and metabolic pathways involved in dyslipidemia during the development of GVHD. There is no widely accepted guideline for lipid-lowering treatment choices for allo-HSCT recipients. In this review, we summarized the pathogenesis of dyslipidemia and focused on the association with GVHD in patients who underwent allo-HSCT. The existing lipid-lowering treatment and promising target agents are also discussed in this review. One limitation in the current study that warrants consideration is that lipid metabolism and the immune response should be discussed in more detail. Due to content limitations, we have not deployed an in-depth discussion on this point in the current review.

Conclusion

Dyslipidemia is a common but underestimated and undertreated comorbidity in allo-HSCT recipients. Long-lasting dyslipidemia will increase the incidence of treatment-related mortality and CVD. The ultimate aim of lowering blood cholesterol levels is to reduce CVD and other events and improve the long-term survival of recipients. Therefore, it is crucial for us to better understand the pathogenesis and explore effective prophylaxis and therapeutic methods for dyslipidemia. In the future, we should pay more attention to the association between dyslipidemia and GVHD. Among the current drugs, statins, fibrates, niacin, and PCSK9 inhibitors might have both lipid-lowering and anti-GVHD effects, and they might be used as promising anti-GVHD candidates. In the end, more laboratory and clinical investigations need to be performed to explore more effective and tolerable strategies to improve dyslipidemia in allo-HSCT recipients.

MS__________

ORIGINALITY REPORT

15%
SIMILARITY INDEX

PRIMARY SOURCES

1	Bernard Lawrence Marini, Sung Won Choi, Craig Alan Byersdorfer, Simon Cronin, David G. Frame. "Treatment of Dyslipidemia in Allogeneic Hematopoietic Stem Cell Transplant Patients", Biology of Blood and Marrow Transplantation, 2015<br>Crossref	130 words — 2%
2	Griffith, Michelle, Madan Jagasia, and Shubhada Jagasia. "Diabetes Mellitus After Hematopoietic Stem Cell Transplantation", Endocrine Practice, 2010.<br>Crossref	80 words — 1%
3	www.bloodjournal.org<br>Internet	80 words — 1%
4	Reena Kumari, Senthilnathan Palaniyandi, Gerhard C. Hildebrandt. "Metabolic Reprogramming—A New Era How to Prevent and Treat Graft Versus Host Disease After Allogeneic Hematopoietic Stem Cell Transplantation Has Begun", Frontiers in Pharmacology, 2020<br>Crossref	59 words — 1%
5	coek.info<br>Internet	56 words — 1%
6	v3r.esp.org<br>Internet	47 words — 1%

7 www.liebertpub.com
Internet
44 words — 1%

8 Melanie Premstaller, Melanie Perren, Kuebra Koçack, Christian Arranto et al. "Dyslipidemia and lipid-lowering treatment in a hematopoietic stem cell transplant cohort: 25 years of follow-up data", Journal of Clinical Lipidology, 2018
Crossref
40 words — 1%

9 qdoc.tips
Internet
35 words — 1%

10 Sophie C. Ragbourne, Martin A. Crook. "Metabolic Syndrome in Long-Term Survivors of Hematopoietic Stem-Cell Transplantation", Clinical Lymphoma Myeloma and Leukemia, 2017
Crossref
34 words — 1%

11 cttjournal.com
Internet
27 words — < 1%

12 www.mdpi.com
Internet
26 words — < 1%

13 www.cureus.com
Internet
24 words — < 1%

14 "Physicians Poster SessionsPoster session / Day 2", Bone Marrow Transplantation, 2016.
Crossref
23 words — < 1%

15 A. Fuhrmann, PC. Lopes, J. Sereno, J. Pedro, D.O. Espinoza, M.J. Pereira, F. Reis, J.W. Eriksson, E. Carvalho. "Molecular mechanisms underlying the effects of cyclosporin A and sirolimus on glucose and lipid metabolism in
21 words — < 1%

liver, skeletal muscle and adipose tissue in an in vivo rat model", Biochemical Pharmacology, 2014
Crossref

16	worldwidescience.org<br>Internet	21 words — < 1%
17	www.nature.com<br>Internet	20 words — < 1%
18	ebin.pub<br>Internet	18 words — < 1%
19	Margaret Vourakis, Gaétan Mayer, Guy Rousseau. "The Role of Gut Microbiota on Cholesterol Metabolism in Atherosclerosis", International Journal of Molecular Sciences, 2021<br>Crossref	15 words — < 1%
20	Michelle L. Griffith, Bipin N. Savani, Jeffrey B. Boord. "Dyslipidemia after allogeneic hematopoietic stem cell transplantation: evaluation and management", Blood, 2010<br>Crossref	14 words — < 1%
21	www.dovepress.com<br>Internet	14 words — < 1%
22	Sheila Doggrell. "Is Atorvastatin Superior to Other Statins? Analysis of the Clinical Trials with Atorvastatin Having Cardiovascular Endpoints", Reviews on Recent Clinical Trials, 2006<br>Crossref	13 words — < 1%
23	Lee, F.Y.. "FXR, a multipurpose nuclear receptor", Trends in Biochemical Sciences, 200610<br>Crossref	12 words — < 1%

24 Peter Sörös, Joachim Böttcher, Hansjörg Maschek, Oliver Selberg, Manfred James Müller. "Lipoprotein-X in patients with cirrhosis: Its relationship to cholestasis and hypercholesterolemia", Hepatology, 1998 — 12 words — < 1%

Crossref

25 bmccomplementmedtherapies.biomedcentral.com — 11 words — < 1%

Internet

26 www.frontiersin.org — 11 words — < 1%

Internet

27 ashpublications.org — 9 words — < 1%

Internet

28 E. Scotti, F. Gilardi, C. Godio, E. Gers, J. Krneta, N. Mitro, E. De Fabiani, D. Caruso, M. Crestani. "Lipid sensing and lipid sensors", Cellular and Molecular Life Sciences, 2007 — 8 words — < 1%

Crossref

29 Lloyd-Jones, Donald M., Pamela B. Morris, Christie M. Ballantyne, Kim K. Birtcher, David D. Daly, Sondra M. DePalma, Margo B. Minissian, Carl E. Orringer, and Sidney C. Smith. "2016 ACC Expert Consensus Decision Pathway on the Role of Non-Statin Therapies for LDL-Cholesterol Lowering in the Management of Atherosclerotic Cardiovascular Disease Risk", Journal of the American College of Cardiology, 2016. — 8 words — < 1%

Crossref

30 Norfarazieda Hassan, Suzy Eldershaw, Christine Stephens, Francesca Kinsella et al. "A single episode of CMV reactivation initiates long-term expansion and — 8 words — < 1%

differentiation of the NK cell repertoire", Cold Spring Harbor Laboratory, 2022
Crossref Posted Content

---

31 Upinder Kaur, Kumudini Acharya, Ritwick Mondal, Amit Singh, Luciano Saso, Sasanka Chakrabarti, Sankha Shubhra Chakrabarti. "Should ACE2 be given a chance in COVID-19 therapeutics: A semi-systematic review of strategies enhancing ACE2", European Journal of Pharmacology, 2020 — 8 words — < 1%
Crossref

---

32 Vinil, Teliki. "A Study on Fasting Lipid Prolife in Non-Diabetic Stroke Patients", Dr. NTR University of Health Sciences (India), 2021 — 8 words — < 1%
ProQuest

---

33 Ying Lu, Junjie Cao, Renzhi Pei, Zhongzheng Zheng et al. "Fludarabine and antithymocyte globulin-based conditioning regimen combined with post-transplantation cyclophosphamide for haploidentical allogeneic hematopoietic stem cell transplantation in patients with high-risk acute myeloid leukemia and myelodysplastic syndrome", Research Square Platform LLC, 2022 — 8 words — < 1%
Crossref Posted Content

---

34 scholar.najah.edu — 8 words — < 1%
Internet

---

35 www.esp.org — 8 words — < 1%
Internet

---

36 www.researchgate.net — 8 words — < 1%
Internet

---

37 "Physicians Poster AbstractsEBMT 2012", Bone Marrow Transplantation, 04/2012 — 7 words — < 1%
Crossref

38 Zufeng Ding, Shijie Liu, Xianwei Wang, Xiaoyan Deng, Yubo Fan, Changqing Sun, Yannian Wang, Jawahar L. Mehta. " Hemodynamic Shear Stress ROS Modulates PCSK9 Expression in Human Vascular Endothelial and Smooth Muscle Cells and Along the Mouse Aorta ", Antioxidants & Redox Signaling, 2015
Crossref

7 words — < 1%

39 "Abstracts of the EASD, Stockholm 2010", Diabetologia, 2010
Crossref

6 words — < 1%

40 "The 46th Annual Meeting of the European Society for Blood and Marrow Transplantation: Physicians Poster Session (P001-P706)", Bone Marrow Transplantation, 2020
Crossref

6 words — < 1%

EXCLUDE QUOTES	ON	EXCLUDE MATCHES	OFF
EXCLUDE BIBLIOGRAPHY	ON		
