# Supplementary material for: Management of dyslipidemia after allogeneic hematopoietic stem cell transplantation
Source: Lipids Health Dis. 2022 Aug 2;21:65. doi: 10.1186/s12944-022-01665-3 (PMC9344644; doi:10.1186/s12944-022-01665-3)

**Randomly selected 5 citations and the original paper with highlighted content**

1. Oudin *et al*. analyzed 170 patients who received allo-HSCT for childhood leukemia. They found that the cumulative incidence of metabolic syndrome (MS) was 13.4% at 25 years and 35.5% at 35 years, which was significantly higher than that among the French population (4% and 5.6%, respectively) [[6](#_ENREF_6)]. （**Page 2-3, Line 35-39）**


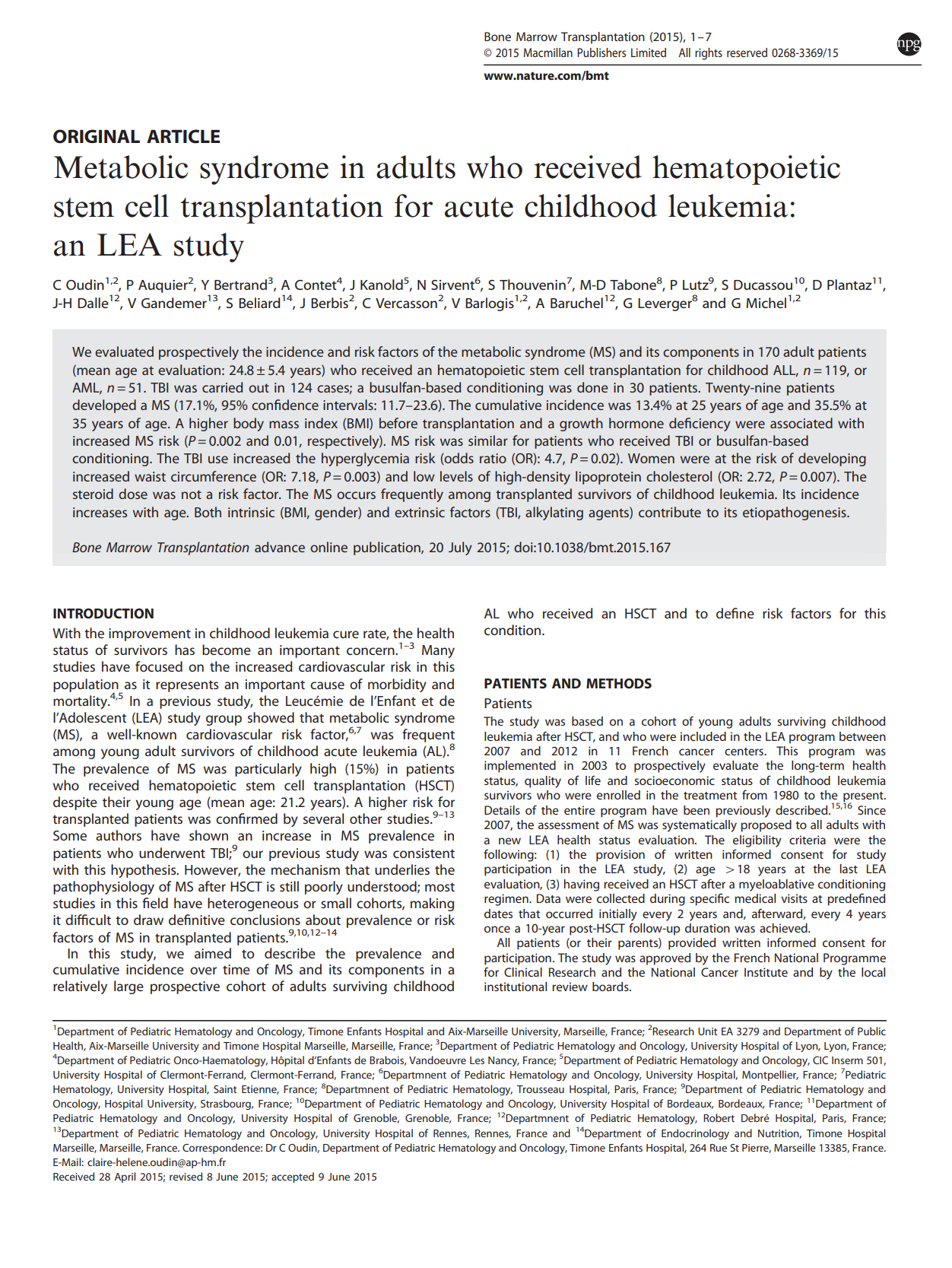
[[6](#_ENREF_6)] Oudin C, Auquier P, Bertrand Y, Contet A, Kanold J, Sirvent N, Thouvenin S, Tabone MD, Lutz P, Ducassou S, et al: Metabolic syndrome in adults who received hematopoietic stem cell transplantation for acute childhood leukemia: an LEA study. Bone Marrow Transplant 2015, 50:1438-1444. https//doi.org/10.1038/bmt.2015.167


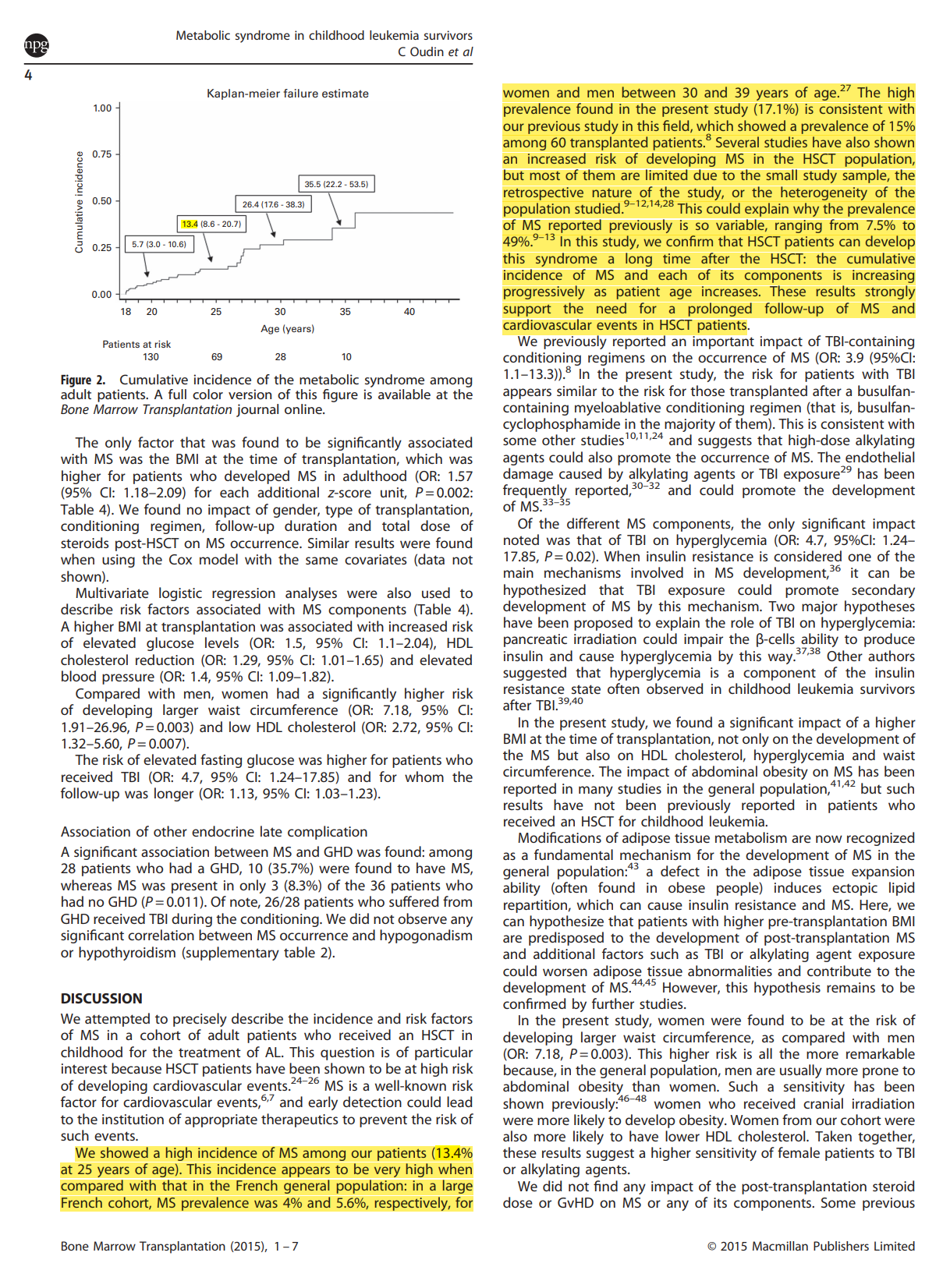


1. During chemotherapy or conditioning, cytotoxic drugs, such as alkylating agents, anthracyclines, antimetabolites, and vinca-alkaloids, can induce mitochondrial dysfunction, and endothelial cytotoxicity leads to insulin resistance, steatosis, and hypertension [[10](#_ENREF_10)]. （**Page 5, Line 89-92）**

[[10](#_ENREF_10)] Rosen GP, Nguyen HT, Shaibi GQ: Metabolic syndrome in pediatric cancer survivors: a mechanistic review. Pediatr Blood Cancer 2013, 60:1922-1928. https//doi.org/10.1002/pbc.24703


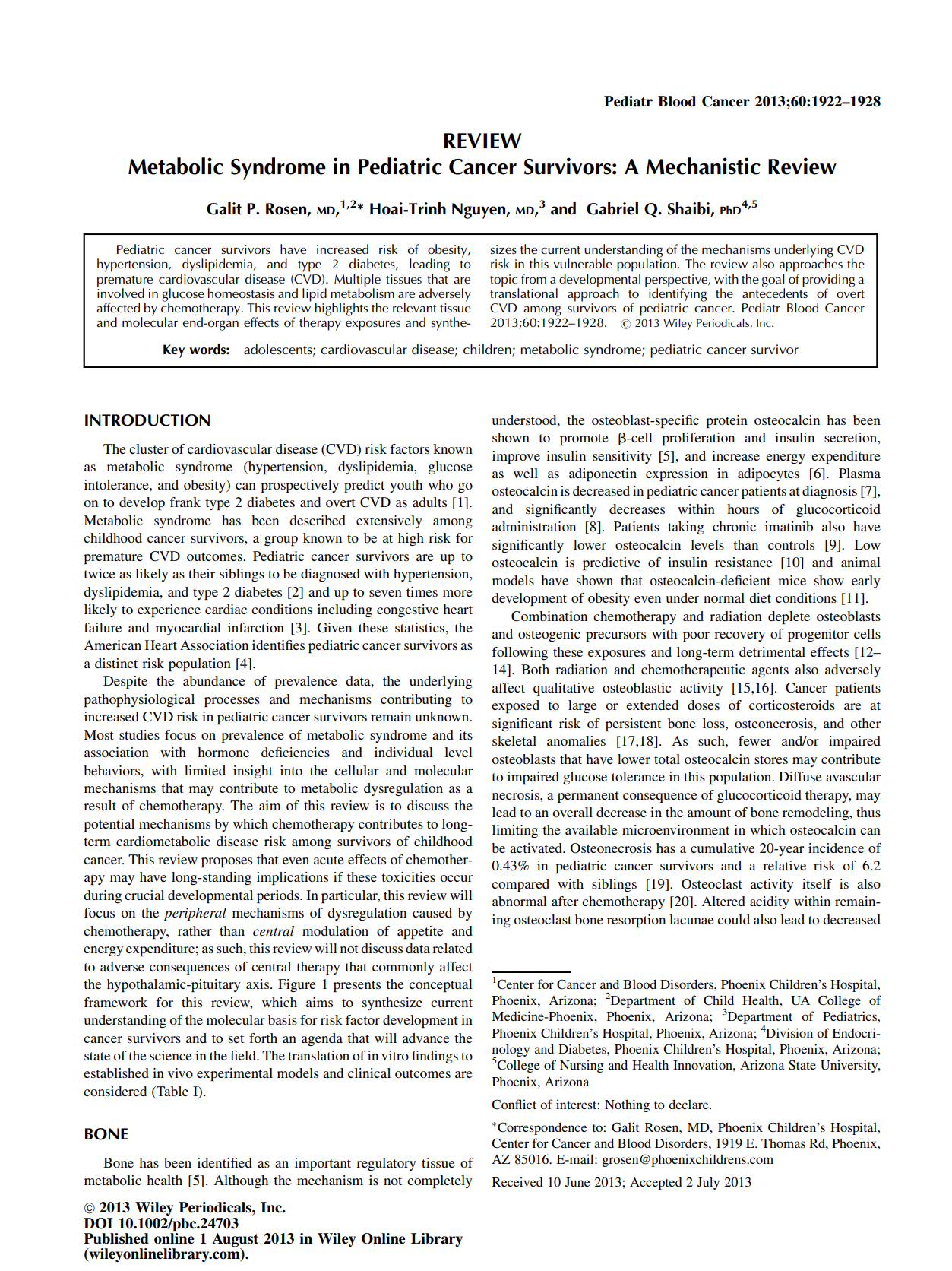


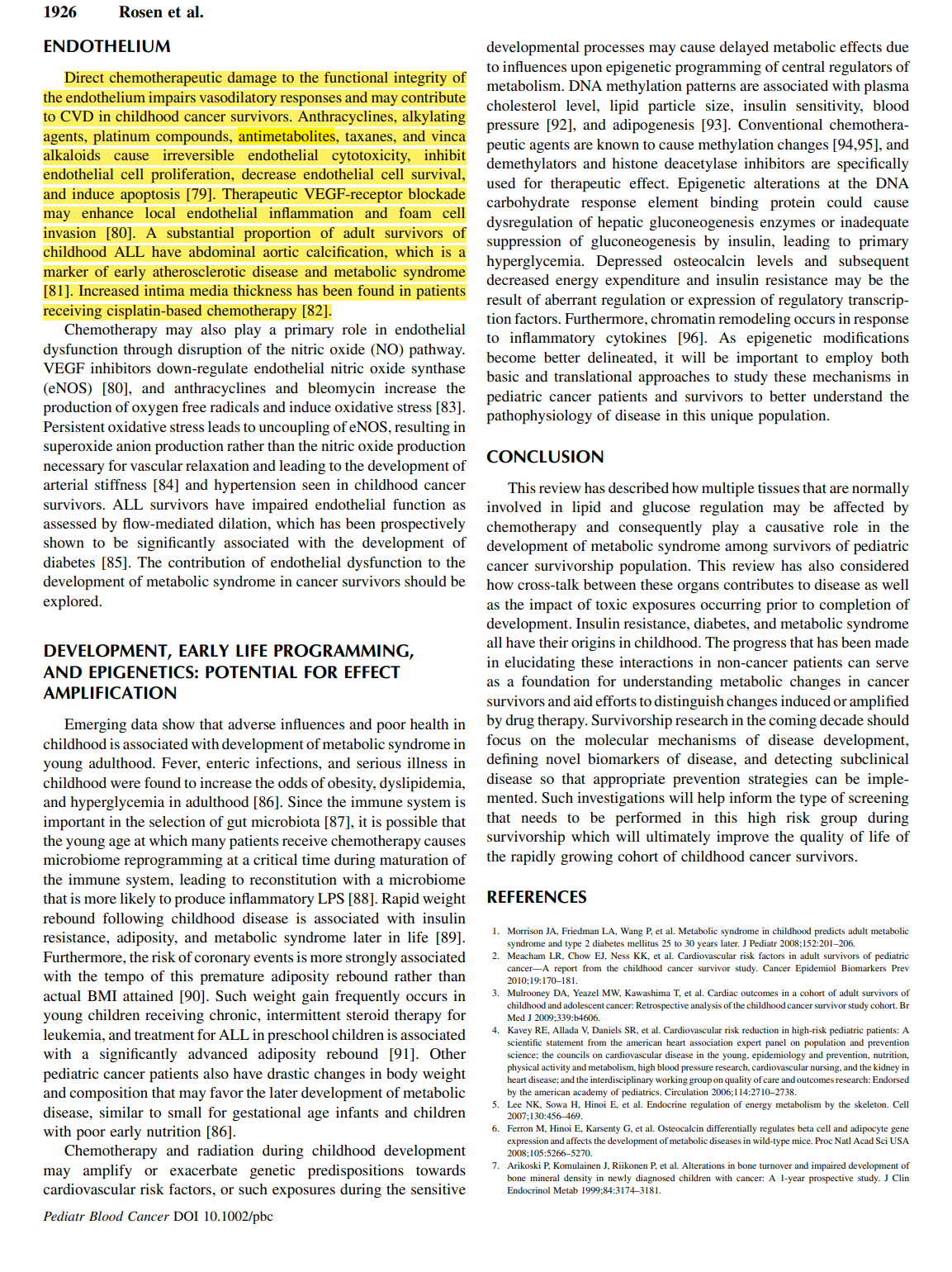


1. Furthermore, LP-X may play a crucial role in the development of cholestatic hypercholesterolemia because it cannot inhibit the synthesis of new cholesterol in the liver [[27](#_ENREF_27)].（**Page 11, Line 195-197）**

[[27](#_ENREF_27)] Soros P, Bottcher J, Maschek H, Selberg O, Muller MJ: Lipoprotein-X in patients with cirrhosis: its relationship to cholestasis and hypercholesterolemia. Hepatology 1998, 28:1199-1205. https//doi.org/10.1002/hep.510280506


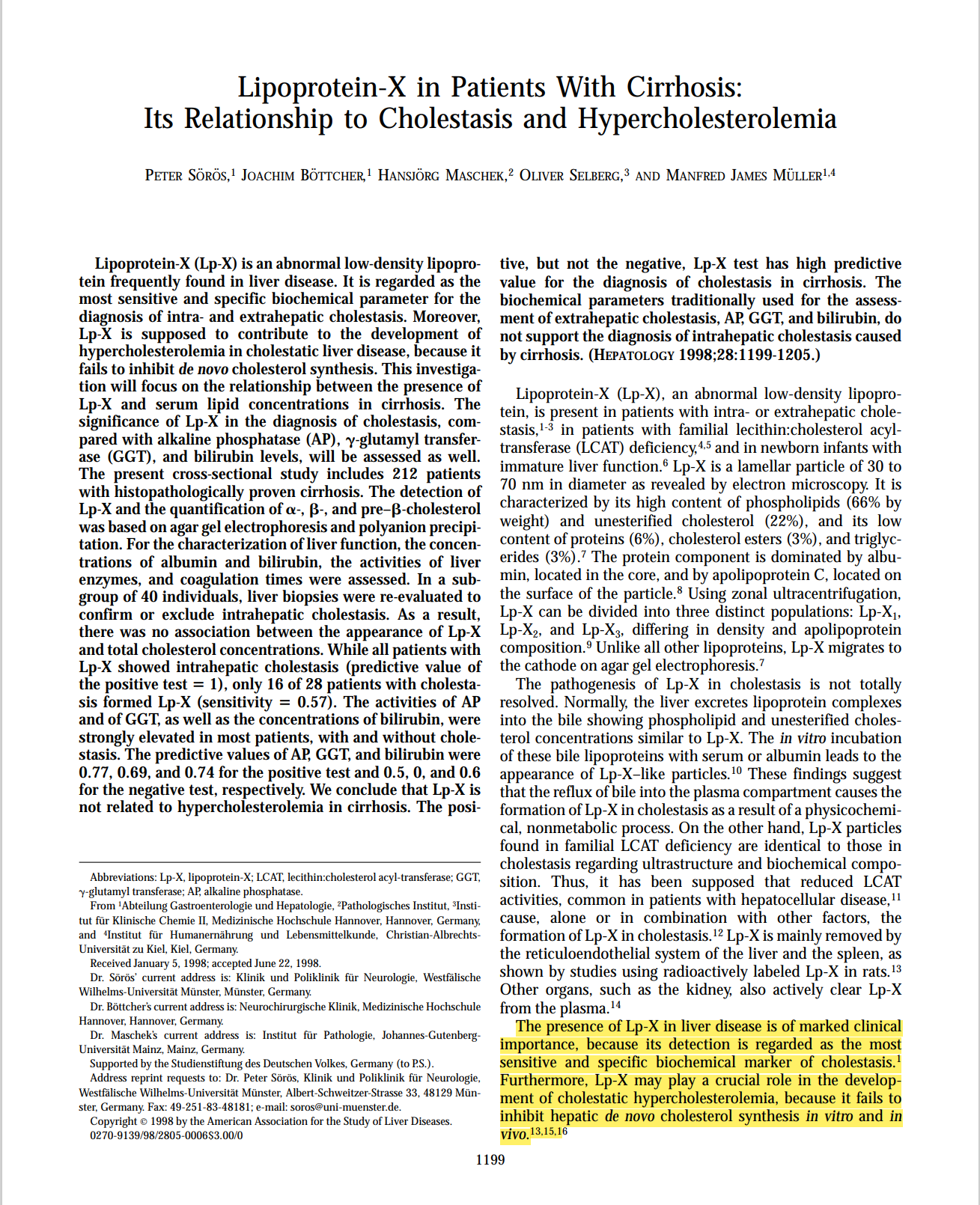


1. Severe mucositis or gastrointestinal complications of GVHD allow many patients to only receive partial or complete parenteral nutrition [[37](#_ENREF_37)]. （**Page 15, Line 257-259）**

[[37](#_ENREF_37)] Bechard LJ, Guinan EC, Feldman HA, Tang V, Duggan C: Prognostic factors in the resumption of oral dietary intake after allogeneic hematopoietic stem cell transplantation (HSCT) in children. JPEN J Parenter Enteral Nutr 2007, 31:295-301. https//doi.org/10.1177/0148607107031004295


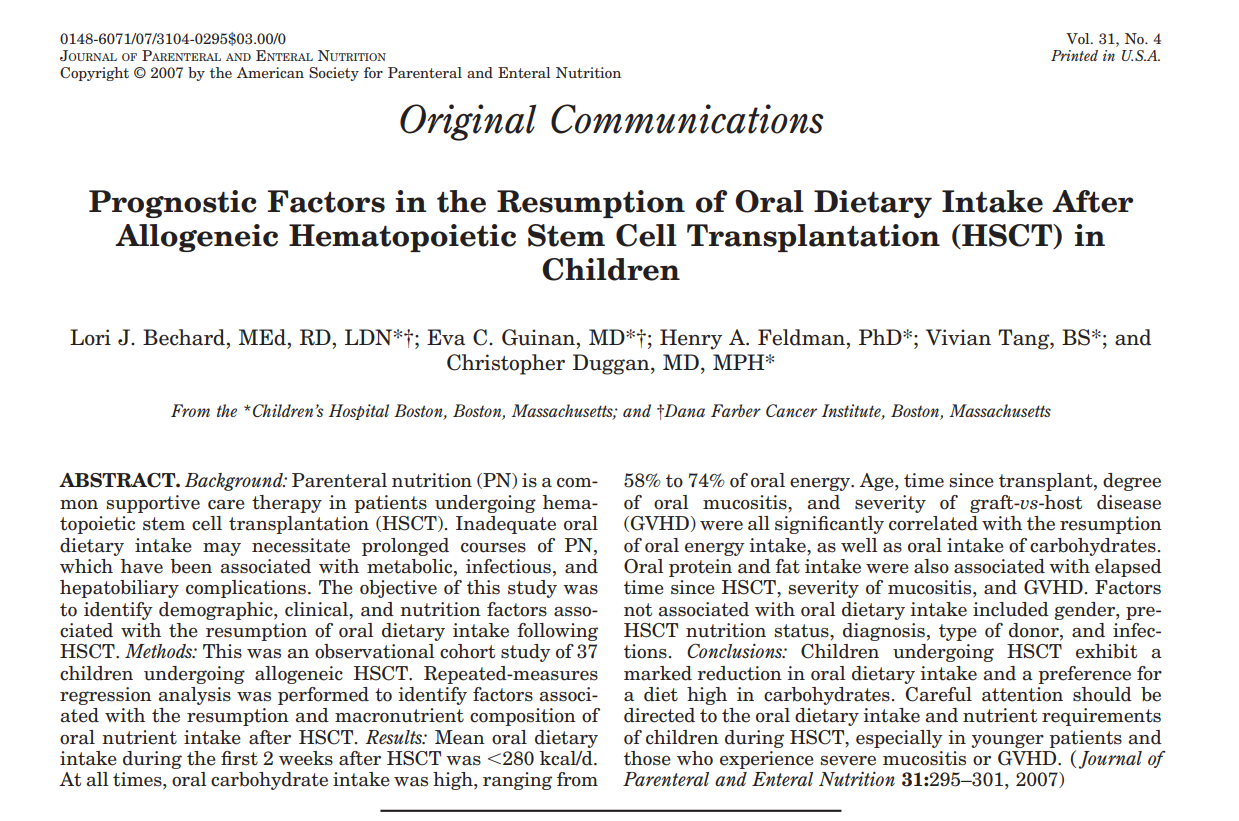


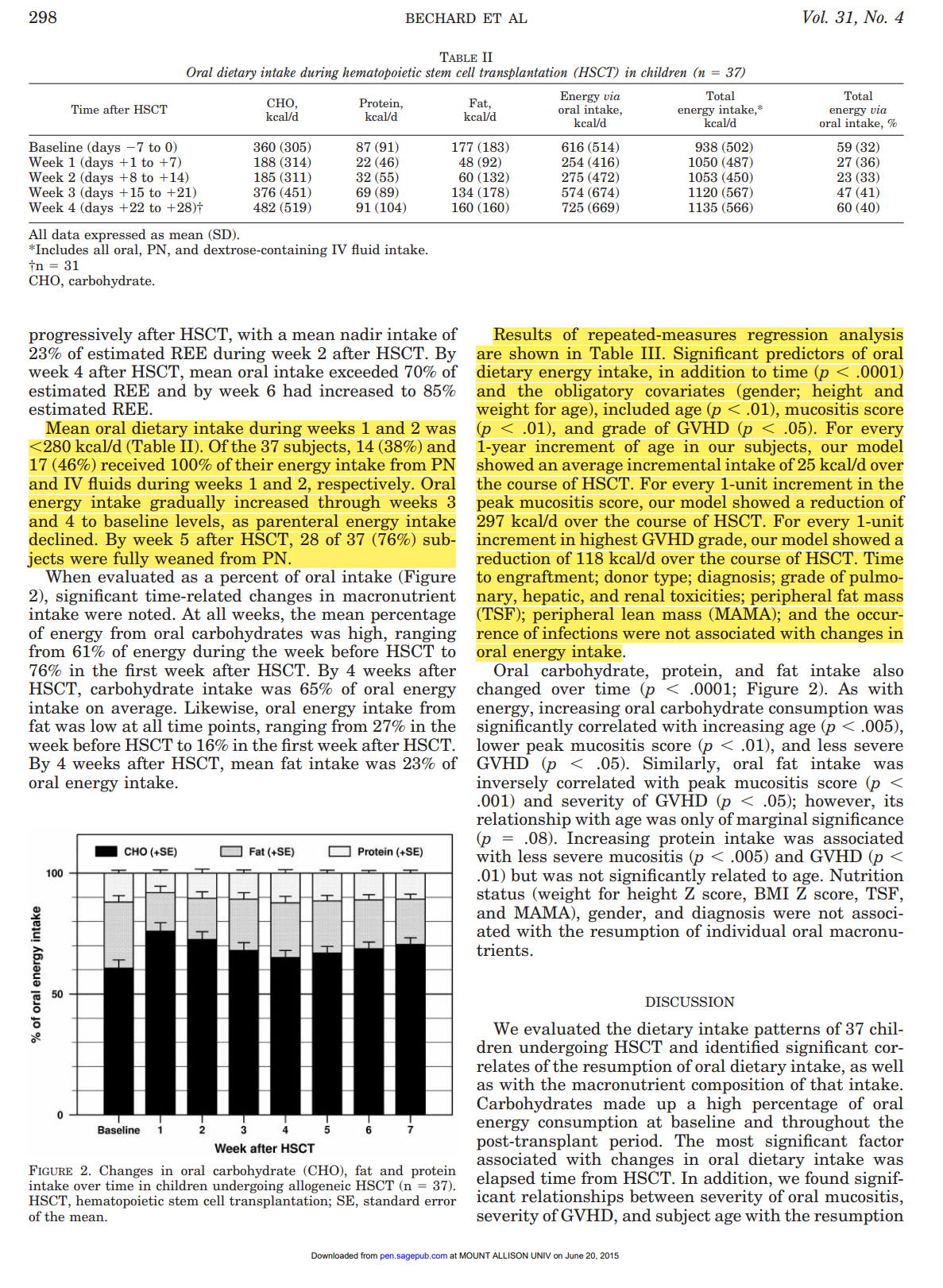


1. Another statin with fewer drug interactions is fluvastatin. It is mainly metabolized by CYP2C9 the pathway (approximately 75%), with a lower percentage being metabolized by the CYP2C8 and CYP3A4 pathways [[43](#_ENREF_43)]. （**Page 21, Line 346-349）**

[[43](#_ENREF_43)] Lawrence JM, Reckless JP: Fluvastatin. Expert Opin Pharmacother 2002, 3:1631-1641. https//doi.org/10.1517/14656566.3.11.1631


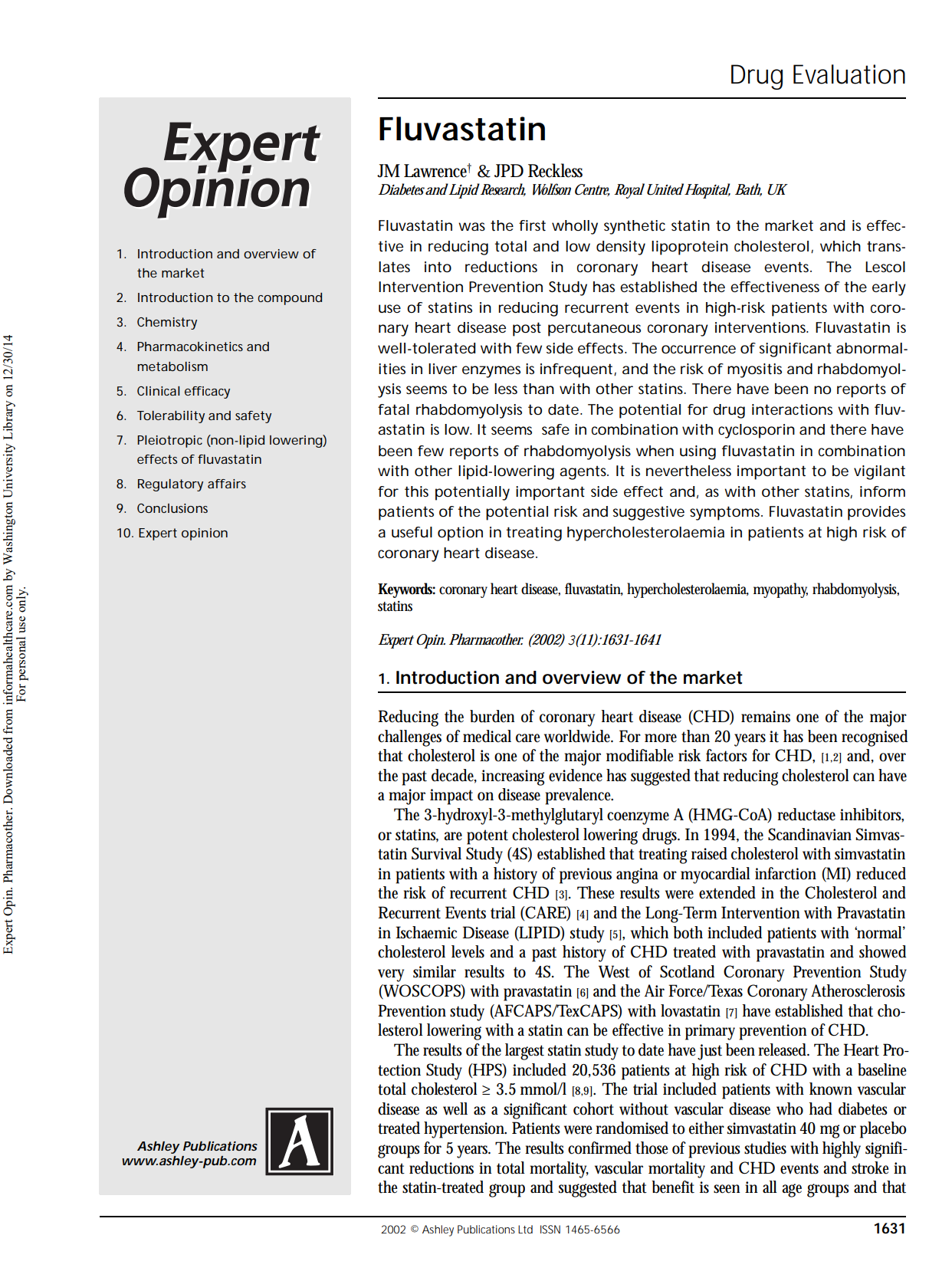


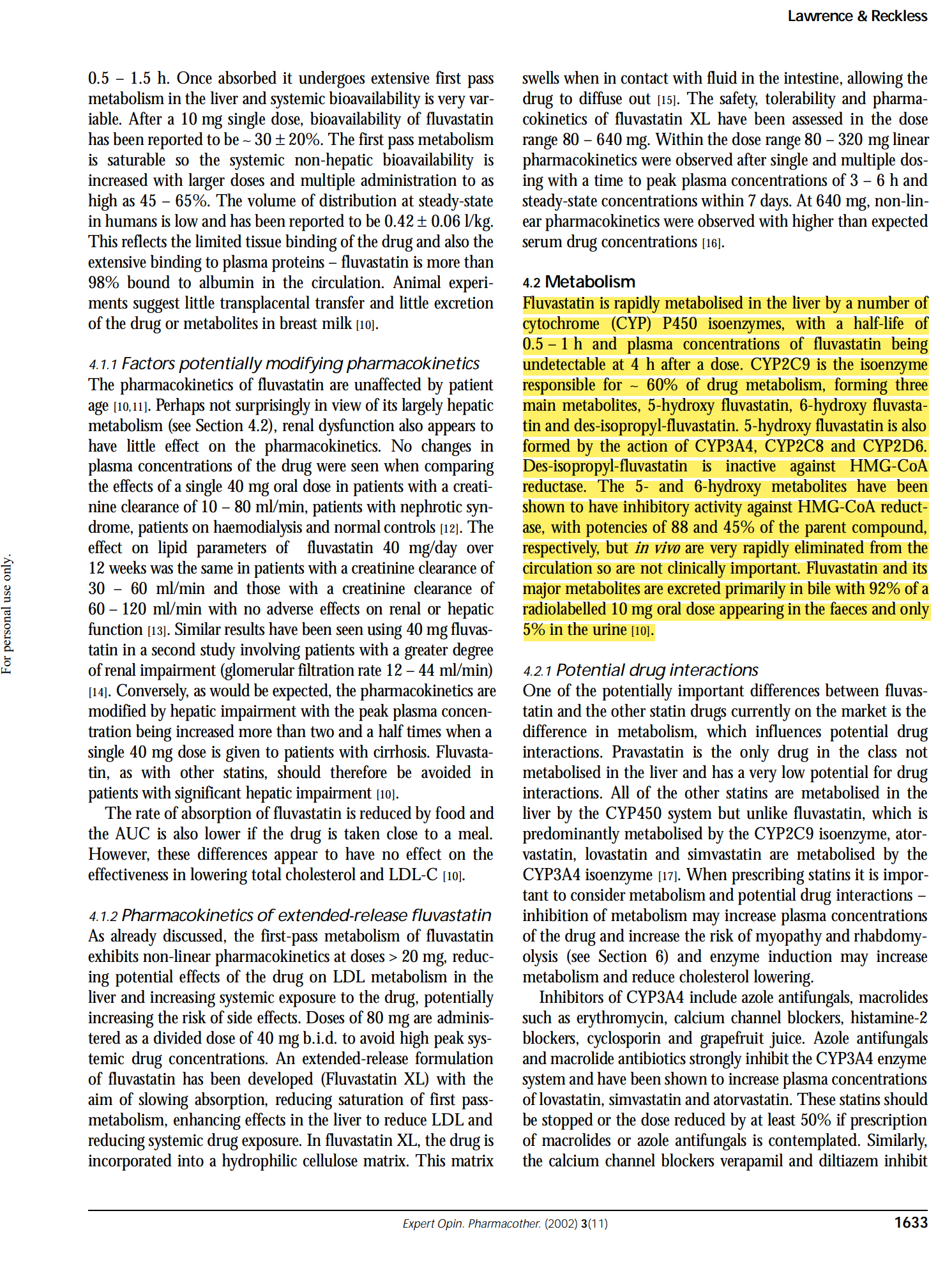

Supplement: Supplementary file 4 — Additional file 4. [file 12944_2022_1665_MOESM4_ESM.docx]
